# School-time physical activity among Arab elementary school children in Qatar

**DOI:** 10.1186/s12887-017-0832-x

**Published:** 2017-03-15

**Authors:** Lena Zimmo, Abdulaziz Farooq, Fuad Almudahka, Izzeldin Ibrahim, Mohamed Ghaith Al-Kuwari

**Affiliations:** 1 Exercise is Medicine, Aspetar, Orthopaedic Sports Medicine Hospital, Doha, Qatar; 2 Athlete Health and Performance Research, Aspetar, Orthopaedic Sports Medicine Hospital, Doha, Qatar

**Keywords:** School children, Physical activity, Moderate-to-vigorous physical activity, Accelerometer

## Abstract

**Background:**

Recent data from a self-administered questionnaire show that approximately 75% of school children in Qatar do not meet the daily recommended levels of physical activity (PA). Since children spend half of their waking hours in school, it is important to understand when and how much PA children accumulate during the school day. This study aimed to objectively assess school-time PA among elementary school children in Qatar and to determine association of PA with age, gender, body mass index (BMI) status, or day of the week.

**Methods:**

A cross-sectional epidemiological study was conducted in four randomly selected elementary schools in Qatar. Two classes representing grade 1 children (age 5) and grade 4 children (age 9) were randomly selected within each school. A total of 183 elementary school children (86 boys and 97 girls) ages 6–12 years participated in this study. PA was assessed using a three-axial accelerometer (ActiGraph® wGT3X-BT). Participants wore accelerometers on their non-dominant wrist at school (7:00 a.m. to 1:00 p.m.) for five consecutive school days during the week. A cutoff points of 818 counts per 5 s was classified as moderate-to-vigorous physical activity (MVPA).

**Results:**

The average duration of MVPA in our study was 28.2 ± 13.5 min per day. Only 39% of participated children reach the recommended school-based MVPA of 30 min or more per day. Students spent on average 58.1 ± 8.4% of school time on sedentary activities. MVPA of boys and girls was similar in age 5 while girls age 9 were less active (23.7 ± 1.5 min/day) than boys of the same age (42.7 ± 1.8 min/day), ES = 0.269, *P <* 0.001. Neither overweight children nor children at risk for being overweight showed any differences in physical activity parameters when compared to children of normal weight. Our results showed, percentage of MVPA on the first (7.7 ± 5.1%) and last (7.1 ± 4.1%) day of the week was generally lower compared to other weekdays (*P <* 0.001).

**Conclusion:**

This was the first study to objectively assess PA during school hours among elementary school-children in Qatar. This study found that many of school children do not perform sufficient time being physically active at school. All students in two age categories (age 5 and age 9) spend the majority of school time engaged in sedentary activities. The low participation of girls age 9 in MVPA is a cause for concern and need to be addressed.

## Background

Physical activity (PA) in children has numerous health benefits. The literature reveals that regular PA of children and adolescent improves cardiorespiratory wellbeing [1], metabolic profile [2], muscular fitness [3] and bone health [4]. Regular PA is also associated with reduced symptoms of depression and stress [5]. Childhood is an important stage of life during which lifestyle behaviors, including PA, are formed and later become established. Active children tend to have active lifestyles in adulthood [6]. The Qatar National Physical Activity Guidelines recommend that children ages 5–11 years participate in at least 60 min of moderate to vigorous physical activity (MVPA) daily [7]. There are no recommendations for school time PA in Qatar. However some countries such as Canada [8] and the United States [9] recommend 30 min of MVPA to be accumulated during the school day.

Despite these facts and recommendations, recent data from a self-administered questionnaire show that approximately 75% of school children in Qatar do not meet the daily recommended PA level [10]. Moreover, about 55% of children spend prolonged periods on sedentary activities such as watching TV and playing video games [10]. The available Qatari data show that there are gender difference in PA practices, with girls engaging in less PA than boys. Studies from Gulf Cooperation Council (GCC) countries [11–18] show that school children meeting 60 min MVPA/day is lower among girls (4 to 39.2%) than boys (43.8 to 70.5%). Although no data exist on changes in PA by age in the GCC area, longitudinal studies show that MVPA consistently declines each year among school children in Western countries [4, 19].

The high prevalence of physical inactivity in Qatar, along with other factors such as poor nutrition, has contributed to a rise in obesity [10]. The prevalence rate of obesity in Qatar is the highest of all GCC countries [20, 21]. Alarmingly, 39% of children in Qatar are classified as overweight, of which 23% are considered obese or morbidly obese [10]. Prospective and cross-sectional studies from the GCC region have found an association of PA with BMI [22] and waist circumference [23] among school-aged children.

There are no existing data on objective measures of PA among children in Qatar. Since, children spend many of their waking hours at school, schools are considered to be an essential setting for children’s PA. An Objective field-based measurement of PA is mandatory to understand when and how much PA is accumulated during the school day among children [24–26]. The aim of this study was the following: 1) to assess objective measures of PA during school hours among elementary school-children in Qatar; 2) to determine if gender, age, BMI status, or day of the week are associated with school time PA.

## Methods

This study was one of the initiatives of Qatar Active Schools (QAS). QAS is a community- based program that aims to enhance the level of PA of every child in Qatar by incorporating PA into the culture of elementary schools and sustaining it through school, family and community partnerships. QAS is guided by Qatar National Vision 2030 which calls for “a healthy population: physically and mentally”([27], p.9), and the Qatar National Health Strategy which aims to prevent chronic diseases through evidence-based programs, including PA programs [20]. The Qatar Active schools program supports action in three zones to create a balanced portfolio of activities that promotes PA in school: a) physical education (PE); b) classroom based physical activity (such as providing PA breaks and adopting physically active teaching method); and, c) physical activity outside class hours (for example, during morning assembly, recess, and extra-curricular periods).

### Study design and setting

This is a cross-sectional study that took place in four primary schools in Doha-Qatar during October-November 2014 period. The schools were nominated by the Ministry of Education and High Education representing Arab and Qatari children in the urban regions. Two were boys-only schools and two were girls-only schools. All schools were similar in terms of size, facilities, socioeconomic status and location (within the capital city of Qatar, Doha). All the schools had indoor and outdoor playgrounds and a large PE arena equipped with the latest sports-related items accessible only during PE class. The classes’ space was relatively large, allowing sufficient space for conducting in-class PA. The outdoor temperature during the data collection period was 28–29 °C and the relative humidity was 54–60%. In all the schools sampled, there were two breaks each day (~25 min in the morning and ~15 min at noon), and the PE classes occurred twice per week (~45 min each).

Two classes representing grade 1 children (age 5) and grade 4 children (age 9) were randomly selected in each school. All students in the selected classes were included in the sample to avoid selection bias within the class. All participants included in the study provided assent and obtained signed parental consent prior to data collection. A sample of 183 (86 boys and 97 girls) out of the 204 school-children were included. From grade 1children, (44 girls and 46 boys) and grade 4 children (53 girls and 40 boys) provided valid data. The grade 1 children ranged in age from 5 to 6 years (5.8 ± 0.4 years) and the grade 4 children from 8 to 11 years (9.0 ± 0.5 years), these groups of children are hereafter referred to as age 5 and age 9 respectively.

### Measurements

Anthropometric data including height and weight were taken. The children wore light-weight school uniforms and were requested to remove their shoes. Height was measured to the nearest 0.1 cm (Seca 242, Germany), and weight was measured to the nearest 0.1 kg using a portable stadiometer (Seca 242, Germany). All anthropometric measurements were recorded by a single staff member in all schools. Participants were either classified as underweight, normal, overweight or obese based on age specific cutoffs as per International Obesity Task Force recommendations [28].

Socio-demographic characteristics such as age, gender, and nationality were determined from school records. Age was calculated based on registered date of birth. Almost all of the participants were Arabs and categorized as Qatari and non-Qatari during analysis.

PA data during school hours were assessed via a tri-axial accelerometer (ActiGraph® wGT3X-BT) capable of processing duration, intensity, and time of PA [29]. Each participant was assigned an accelerometer to record PA only during school hours (from 7:00 a.m. to 1 p.m.) over five consecutive days of the school week. The accelerometers were strapped around the non-dominant wrist of each participant by a pre-trained teacher as soon as students arrived in class and were removed and collected at the end of the school day. The decision to place the accelerometer on the wrist instead of the hip was made for practical reasons. The school uniform for boys is a long white traditional dress (*Thaub*) and for girls a similar long one-piece dress. Neither garment has a belt holder in which to place an accelerometer. In children of similar age groups, wrist-based accelerometers have shown good compliance [30, 31]. Moreover, a recent study of children ages 8–12 years confirmed that wrist-worn accelerometers can provide meaningful PA intensities in free-living conditions [32].

The school children in our study were instructed not to remove their wrist-worn accelerometers under any circumstances except if they were finding them uncomfortable. The accelerometers did not have to be removed for other reasons since no swimming or water activities were planned in school hours during the data collection period. The monitors were initialized to begin data collection on Sunday at 7:00 a.m. and end on Thursday at 12:00 noon, with an epoch length of 10s. The software identified non-wear time as an interval of at least 10 min of null activity readings, with 120 consecutive seconds of counts less than 100. We only included school time data since there were no planned after school activities during the study period. Students with at least four hours of valid wear-time data for three or more days were included in the analysis. PA was determined as the total count of activity divided by the total duration of wear time to determine counts per minute (CPM). Time spent doing sedentary, light, moderate, and vigorous activity was classified for each student using VM cutpoints as follows: sedentary (<305 counts per 5 s); light (≥306 to ≤817 counts per 5 s); moderate (≥818 to ≤1968 counts per 5 s) and vigorous (≥1969 counts per 5 s). Activity was classified as MVPA when VM was > =818 counts per 5 s [32].

### Statistical analysis

All data were coded and analyzed using Statistical Package of Social Sciences (SPSS) v21.0. Descriptive summary statistics of continuous variables were presented as mean ± standard deviation (SD). A chi-square test was used to compare the distribution of age groups and nationality groups among boys and girls. A student *t*-test was used to compare mean age and anthropometric data between boys and girls across each age group. General linear models were used to determine the effect of factors such as gender, age group (age 5 vs. age 9), nationality group (Qatari vs. Non Qatari), and BMI status on PA measured as vector magnitude (cpm). Similar general linear models were performed to determine the effect of gender and age group on the average MVPA in min/day and the average percentage of time spent on sedentary, light, moderate, and vigorous PA. To determine the effect of day of week (Sun to Thu) on PA, measured as vector magnitude (cpm), linear mixed models were performed that accounted for repeated measurements with an unstructured covariance type. Partial eta-squared was used to represent effect sizes (ES), 0.01 being small, 0.06 medium and 0.14 large. *P-*value <0.05 was the cutoffs for reporting statistical significance.

## Results

A total of 183 children (97 girls and 86 boys) provided valid accelerometer data for total school time during a week. Table 1 shows the age and anthropometric characteristics of the participants by gender. The sample included equal proportions of boys and girls representing children in two age categories: age 5 and age 9. There were no differences in body mass index between boys and girls for each age group category. The majority of the participants 132 (72%) were Qatari children. However, the proportion of non-Qatari girls (76.5%) participating in this study was higher than Qatari girls (43.9%, *P <* 0.001).

**Table 1:** Characteristics of the participants (*N =* 183)

Variable	Girls *n =* 97	Boys *n =* 86	*p*-value
	n (%)	n (%)	
Grade			
One (age 5)	44 (48.4)	47 (51.6)	0.210^a^
Four (age 9)	53 (57.6)	39 (42.4)	
Nationality			
Qatari	58 (43.9)	74 (56.1)	<0.001^a^
Non Qatari	39 (76.5)	12 (23.5)	
Anthropometry	Mean ± SD	Mean ± SD	
Grade One			
Age (Years)	5.7 ± 0.5	5.9 ± 0.6	0.040^b^
Height (cm)	118.1 ± 5.8	119 ± 10.5	0.652^b^
Weight (Kg)	22.9 ± 5.8	22.4 ± 4.6	0.601^b^
Body mass index (Kg/m^2^)	16.2 ± 2.9	15.9 ± 2.6	0.500^b^
Grade Four			
Age (Years)	9.1 ± 0.6	8.8 ± 0.4	0.010^b^
Height (cm)	136.9 ± 7.9	133.7 ± 8.3	0.063^b^
Weight (Kg)	37.2 ± 13.8	36.6 ± 11.9	0.834^b^
Body mass index (Kg/m^2^)	19.6 ± 5.7	20.1 ± 4.6	0.626^b^

^a^A chi-squared test for homogeneity

^b^Student *t*-test

Table 2 shows the observed average PA (mean ± SD) expressed as vector magnitude counts per minute (CPM) for boys and girls separately. The pairwise comparisons were based on differences in estimated marginal means from the general linear model, which included gender, age group, BMI status, and nationality groups. The PA was generally higher among boys (4153 ± 881) than girls (3537 ± 710), ES = 0.770, *P <* 0.001). Boys and girls age 5 showed similar levels of PA during school time (*P =* 0.597), whereas boys age 9 (4635 ± 919) were more physically active than girls of the same age (3473 ± 744; ES = 0.143, *P <* 0.001). Qatari girls were less active (3578 ± 732) than Qatari boys (4155 ± 905; ES = 0.089, *P <* 0.001). However, physical activity levels were similar in non-Qatari boys and girls (*P =* 0.139).

**Table 2:** Physical activity measured as vector magnitude counts per minute (CPM) in boys and girls

Variable	Girls *n =* 97	Boys *n =* 86	Difference	Effect size	*P*-value
	Mean ± SD	Mean ± SD	Mean (95% CI)		
	3536.5 ± 710.3	4152.8 ± 880.8		0.770	<0.001
Grade					
One	3613.5 ± 668	3752.4 ± 611.6	−129 (−351, 608)	0.002	0.597
Four	3472.6 ± 743.9	4635.4 ± 919.3	−1134 (−1575, −693)	0.143	<0.001
Nationality					
Qatari	3578.1 ± 732	4155.3 ± 905.2	−765 (−376, −1153)	0.089	<0.001
Non Qatari	3474.6 ± 681.4	4137.6 ± 745.9	−452 (−148, 1052)	0.014	0.139
Overweight Status					
Underweight	3103.2 ± 784.6	3994.8 ± 384.5	−771 (−1774, 232)	0.015	0.131
Normal	3698.4 ± 648.9	4019.5 ± 898.3	−561 (−929, −194)	0.056	0.003
Overweight	3364.8 ± 679.6	4242.1 ± 847.7	−420 (−1128, 287)	0.009	0.243
Obese	3547.2 ± 724.7	4521.8 ± 876	−742 (−1400, −83)	0.031	0.027
Day of the week					
Sunday	3393.9 ± 873.0	3708.9 ± 1302.5	−352 (−675, −29)	0.025	0.033
Monday	3468.5 ± 736.4	4693.6 ± 1088.8	−1258 (−1560, −956)	0.291	<0.001
Tuesday	3762.7 ± 675.9	4462.3 ± 1136.7	−744 (−1040, −448)	0.124	<0.001
Wednesday	3814.7 ± 892.6	4015.7 ± 928.1	−166 (−434, 103)	0.008	0.224
Thursday	3384.8 ± 1042.2	3694.4 ± 1147.9	−315 (−636, −6)	0.020	0.054

Overall PA (cpm) was not associated with BMI status in boys and girls (Table 2). However, both normal weight and obese boys were generally more active than obese girls (*P =* 0.003 and *P =* 0.027, respectively). The combined activity of boys and girls during the first day (Sun) and last day (Thu) of the school week was significantly lower than their activity on other school days (Mon, Tue and Wed). Boys were more active than girls on the first three days of the week only (*P <* =0.033).

**Fig. 1:**
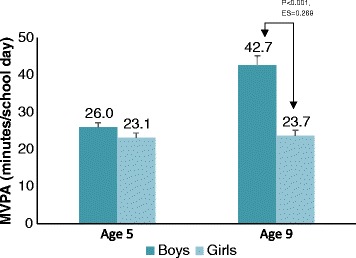
Estimated marginal means (±SE) of Moderate to vigorous physical activity (MVPA) during school day by grade and gender

**Fig. 2:**
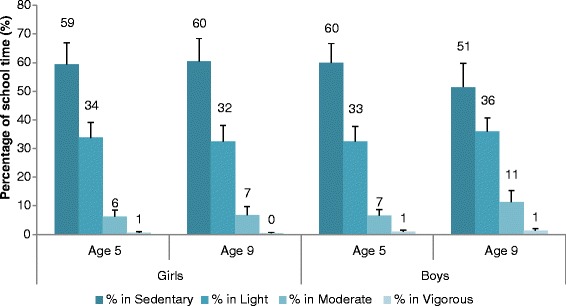
Average proportion of the day (Mean ± SD) representing breakdown of physical activity levels during school time by age group and gender

The MVPA of age 5 children was estimated to be an average of 26.0 ± 8.6 and 23.1 ± 8.8 min per day for boys and girls, respectively, but this was not statistically different. On the other hand, age 9 boys had higher MVPA (42.7 ± 16.1 min per day) when compared to girls of the same age (23.7 ± 10.3 min per day, ES = 0.269, *P <* 0.001) (Fig. 1). Figure 2 shows that both boys and girls spent a very small proportion of school time taking part in vigorous activities (<2%) and that more than half of the school day was spent on sedentary activities.

## Discussion

This was the first study to objectively assess PA among school children in Qatar during school time. In other regions, few studies have focused on school-time PA alone [33], and not all of them have analyzed the differences in day of the week as a factor to describe MVPA [31].

In our study, the average MVPA assessed by wrist accelerometer was 24.5 ± 8.7 min/day among age 5 children and 31.8 ± 16.1 min/day among age 9 children. These levels of PA were higher than those in studies using hip-mounted accelerometers. For example, a study of age 10 children (10.4 ± 0.3 years) from North-West England found that the average MVPA during school time was 28.4 and 23.3 min among students classified as high active and low active, respectively [34]. Among school children (10–14 years), the average school time MVPA was reported to be 19.6 ± 0.6 and 19.0 ± 0.7 min among school children from Liverpool and Madrid, respectively [35]. In a European study of 14 year olds, the average school time MVPA was 23.0 ± 0.59 min for Spanish children and 28.5 ± 0.56 min for French school children [36]. However, the breakdown of physical activity levels in our study indicated that participation in vigorous physical activity was very minimal (Fig. 2).

Our study reports that a great amount of time was spent in sedentary activities. It appears that traditional teaching methods are still being used. There is a need to introduce physically active teaching methods. Our study showed that during a six-hour school day, the average duration in sedentary time was 194.8 ± 38 min. This is relatively high when compared to a study that found in a seven-hour school day, children can spend between 150 and 200 min in sedentary activities [35]. This difference is a cause for concern and signifies the need to re-evaluate the non-PE classes, in which children are more likely to be sedentary.

The lower physical activity and higher sedentary behavior among girls compared to boys of similar age has been well documented among several populations in Europe [37, 38], the United States [39], and Australia [40]. Whereas results from these reports suggest that differences among the PA of boys and girls are seen at all ages, our data showed that physical activity among boys and girls were similar in age 5 children. However, when it comes to older children, our results correspond to those of other studies: greater activity was seen among age 9 boys than among age 9 girls. The wide difference in MVPA among age 9 boys and girls is a cause for concern and suggests that greater declines in a girl’s physical activity may occur with maturity.

The physical education curriculum in all schools is the same for each grade irrespective of gender. However, since the schools in our study were separated by gender, it was not possible to ascertain whether a similar PE curriculum was delivered in both the boys’ and girls’ schools. The lack of facilities cannot be an issue since all schools were spacious and well equipped. Despite the availability of facilities, however, the children were mostly restricted to indoor playing areas. The reasons are still not clear whether the choice of indoor space was due to school policy or to instructor preferences. Therefore, it is essential to further investigate and determine distribution of physical activity intensity during physical education, recess, and breaks.

Our study showed no correlation of physical activity with BMI status. Conversely, Page et al., 2005 [41], showed obese (>99th Percentile) children (age 10 years) had lower volume and intensity of physical activity compared to non-obese children. Extreme obesity can be a barrier to physical activity, but in children BMI does not often correlate with low physical activity [42]. There is definitely a large area of further research to be done in these settings by taking socio-ecological and organizational factors into consideration.

In the Gulf region, the usual school exposure time is from 7:00 a.m. to 1:00 p.m. (six hours) Sunday to Thursday. Based on our study, we discovered that PA is lower on the first and last days of the school week. This finding was similar in age 5 and age 9 children. The low activity during the last day of week (Thursday) can be explained by a shorter time in school on that day (about 45 min less). However, significantly lower PA on the first day cannot be explained by our data. Other studies have found greater MVPA on the first day of the school week, but these studies measured whole day-activity rather than only school-time activity [31]. To explain the low PA on the first school day of the week, we would have to study the activity during the weekend. Explanations could either be related to unorganized sleeping habits or to extra activity during the weekend. The total sleep time during weekends may be higher [43, 44], but the usual bedtime at weekends among elementary school children is reported to be 1 h 12 m later than weekday bedtimes [43]. As a result, we believe that children may be sleep deprived on the first day of the week, but since we have neither weekend activity nor sleep data to support this hypothesis, further research in this area is needed.

The physical activity was measured during the October-November months in Qatar when the average outdoor temperature and humidity is 28 °C - 29 °C. We expect hot weather to be a barrier; therefore, more studies of longitudinal design are needed to determine seasonal variation and patterns in physical activity among school children.

The primary limitation of this study was that the objective measures of physical activity were based on wrist-worn accelerometers. Because of this, our data might not be comparable with other studies that generally use hip-worn accelerometers. However, in the current setting, where the school uniform is the traditional long *thoub* for boys and a long skirt for girls, hip-worn accelerometers cannot easily be attached to their garments. The advantage of using the wrist-worn accelerometer is that it increases compliance and convenience [30, 31]. There are only a handful of studies that describe the classification of physical activity intensities based on wrist-worn accelerometers in young children [32, 45, 46]. In this study Chandler et al. [31] cutoffs were used because they represented ages 8 to 12 years when the accelerometer was positioned on the non-dominant wrist. On the other hand, Crouter et al. [31] validated cutoffs for MVPA on the dominant wrist. In earlier research by Ekblom et al., cutoffs could not be used in our study because a different brand of accelerometer (Actiwatch) was used to the one used in our study (Actigraph).. Applying the Crouter et al., cutoffs, the overall prevalence of MVPA during school time in this study was 17.0% compared to Chandler at 8.5%. The wide difference between the two results suggests the need for more validation studies on wrist-worn accelerometers. The other limitation is that this study focused on only children in two specific age groups: age 5 and age 9. We intended to target children in the early years of elementary school and mid-years of elementary school education. Moreover, these age groups have been targeted by several studies on school children [31, 33, 35, 47–50]. An additional limitation to this study is that we only collected school-time physical activity. This was done in order to avoid low compliance and loss of activity monitors. The compliance on valid wear-time physical activity is lower in children of this age group [51], and there is an additional risk of the device being lost or returned late. Our study showed high compliance with 183 of the 215 children (85.1%), all of whom received consent and wore the accelerometer for at least more than three days of the week.

## Conclusion

This is the first study to objectively assess PA during school hours among elementary school-children in Qatar. This study highlights three major areas that need immediate attention. All students in two age categories (age 5 and age 9) spend the majority of school time engaged in sedentary activities. The low proportion of time spent on vigorous activity necessitates the need to re-evaluate the physical education in elementary school children in Qatar. The MVPA in age 9 girls was lower than age 9 boys, suggesting earlier decline than expected among girls.

## Abbreviations

BMI: Body mass index
CDC: Center of Disease Control
CPM: Counts per minute
MVPA: Moderate-to-vigorous physical activity
PA: Physical activity
PE: Physical education
QAS: Qatar Active Schools
SPSS: Statistical package of Social Sciences
VM: Vector magnitude
